# Understanding Family Functioning as a Protective Factor for Adolescents’ Mental Health from the Parental Perspective: Photovoice in Rural Communities of Ecuador

**DOI:** 10.3390/ijerph22101471

**Published:** 2025-09-24

**Authors:** Venus Medina-Maldonado, Majo Carrasco-Tenezaca, Molly Frey, Esteban Baus-Carrera

**Affiliations:** 1 Centro de Investigación para la Salud en América Latina (CISeAL), Faculty of Health and Wellness, Nursing School, Pontifical Catholic University of Ecuador, Nayón 170530, Ecuador; vemedinam@puce.edu.ec; 2 Centro de Investigación para la Salud en América Latina (CISeAL), Faculty of Habitat, Infrastructure and Creativity, Architecture School, Pontifical Catholic University of Ecuador, Nayón 170530, Ecuador; mjcarrasco@puce.edu.ec; 3 Heritage College of Osteopathic Medicine (OU-HCOM), Ohio University, Dublin, OH 43016, USA; mf148021@ohio.edu; 4 Centro de Investigación para la Salud en América Latina (CISeAL), Observatorio de Comunicación (OdeCom), Faculty of Health and Wellness, Medicine School, Pontifical Catholic University of Ecuador, Nayón 170530, Ecuador

**Keywords:** adolescent mental health, rural communities, photovoice, protective factors

## Abstract

Families in rural communities face a constellation of challenges that significantly hinder their ability to support adolescents. Our study aimed to explore family functioning as a protective factor for adolescent mental health from the perspective of parents in rural communities of southern Ecuador, using Photovoice as a participatory research tool. The research design corresponds to Participatory Action Research. Five Focus Group Discussions (FGDs) were conducted. A total of 29 parents of adolescents participated in the study. The research team employed qualitative content analysis for the interpretation phase. Through photographs and focus groups, parents commented on aspects of family life that they perceived as necessary for supporting adolescents, such as effective communication, cohesion, supervision, and expressions of care. The main conclusion indicated that the implementation of Photovoice converted participants from subjects to collaborators, allowing them to critically reflect on their behaviors while aiding or reinforcing in the co-creation of strategies.

## 1. Introduction

Mental health is universally acknowledged as a fundamental human right and a cornerstone of sustainable development. The Lancet Commission on Global Mental Health and Sustainable Development emphasizes that mental well-being is not only a determinant of individual quality of life but also a driver of economic growth and societal progress [1,2]. However, in rural settings, particularly in low- and middle-income countries (LMICs), this ideal remains distant. Adolescents in these regions encounter structural barriers that severely restrict access to mental health services. Inadequate health infrastructure and a chronic shortage of trained professionals create significant service gaps. Deeply entrenched socioeconomic disparities further amplify their vulnerability. Yet, within these challenging contexts, families and communities serve as powerful protective forces. These strengths, embedded in the social fabric, provide critical emotional and psychological support, buffering against the risks associated with the lack of specialized care [3,4].

Adolescent mental health—especially in the scope of community nursing—presents a profoundly complex challenge. It emerges from a web of interdependent factors that shape emotional well-being and requires approaches that are both holistic and precise. Community nursing, by its nature, engages with the nuanced interactions between individuals, families, and the broader community. Social and familial environments exert a decisive influence, either fortifying mental health or exacerbating its fragility [5,6]. Central to this dynamic are protective factors: open communication that fosters connection, emotional support that anchors stability, and parental supervision that guides without constraining. These factors not only counteract the corrosive impact of risk but also cultivate resilience. For adolescents, they serve as both refuge and strength—a duality that transforms vulnerability into growth. Ultimately, such factors do more than shield; they fortify, enabling young people to navigate adversity and emerge with a reinforced sense of self [7,8].

Community nursing stands at the crossroads of these challenges, with a role as multifaceted as the issues it addresses. Adolescent mental health is shaped by an intricate interplay of individual, family, and community factors. In this context, community nursing functions not as a reactive service but as a proactive bridge, weaving these dimensions together to promote well-being. The influence of family and social environments—widely recognized in the academic literature—is fundamental [9,10]. Families strengthened by open communication, consistent emotional support, and supervision that empowers rather than restricts are better equipped to help adolescents face adversity and build lasting resilience [11,12].

Families in rural communities face a constellation of obstacles that undermine their ability to support adolescents. Economic hardship compounds geographic isolation, limiting access to education and mental health services [13]. These are not merely logistical barriers but structural inequities that entrench vulnerability. Even so, opportunities for transformation exist. Through education and intervention, community nursing serves as a bridge that connects families with essential services and empowers them to act. By fostering locally driven strategies tailored to community needs, nurses help families transcend environmental constraints, build resilience, and strengthen their capacity to nurture adolescent development [14].

Our study addresses this issue through an innovative lens, employing Photovoice to explore parents’ perceptions of family strengths as protective factors for adolescent mental health [15]. Photovoice is more than a research tool—it is a participatory process that combines the evocative power of photography with the depth of personal narratives. It allows participants to reflect on and communicate their lived experiences visually and verbally, while also serving as a platform for empowerment by positioning parents as co-creators in the research process [16]. In doing so, it bridges research and action, ensuring that solutions are both contextually relevant and rooted in the communities they serve [17].

In the rural communities of Loja, a province in southern Ecuador, family cohesion is not merely supportive—it is foundational to adolescent mental health. Parents identify protective factors such as effective communication that fosters understanding, expressions of affection that nurture emotional security, supervision that provides guidance without overcontrol, and unwavering emotional support. These pillars anchor resilience and safeguard well-being [18,19]. Nevertheless, families simultaneously face persistent economic constraints, geographic isolation, and a lack of access to mental health training programs [15,20]. These challenges are systemic, compounding the vulnerabilities of rural life. Addressing them requires more than conventional interventions; it demands contextually grounded strategies informed by the lived experiences and resources of the community itself. Only by integrating such perspectives can mental health initiatives become both effective and sustainable.

Although adolescent mental health in rural settings is widely recognized as a public health concern, few studies have examined how families in LMICs contribute to protecting adolescent well-being. Most existing research has focused on risk factors and service deficiencies, overlooking the protective role of family functioning and parental perspectives. Furthermore, there is limited evidence from participatory qualitative approaches such as Photovoice, which allows parents to express their lived experiences and identify culturally relevant strategies to strengthen adolescent mental health. This gap is particularly evident in rural Ecuador, where geographic isolation, poverty, and limited access to care pose additional barriers. Addressing this gap is essential for designing family-centered, community-led interventions adapted to these contexts.

The aim of this study was to explore family functioning as a protective factor for adolescent mental health from the perspective of parents in rural communities in southern Ecuador, using Photovoice as a participatory research tool.

This article builds directly on our previous study, Risk Factors for the Mental Health of Adolescents from the Parental Perspective: Photovoice in Rural Communities of Ecuador [15], published in this journal in 2023, providing complementary insights into resilience in rural contexts. Both studies were conducted in June 2022 with the same participants. While the earlier study identified risk factors for adolescent mental health, the present article advances this work by examining family functioning as a protective factor. Together, these complementary analyses provide a more holistic understanding of vulnerabilities and strengths in rural families, offering insights to guide culturally relevant, community-based interventions.

## 2. Materials and Methods

### 2.1. Design

The research design was based on Participatory Action Research (PAR). Five Focus Group Discussions (FGDs) were conducted to explore the meanings, aspirations, values, and perceptions of parents regarding family functioning as a protective factor for adolescents’ mental health. As in all PAR processes, the research team played an active role in guiding group reflections, which may have influenced participants’ responses. To mitigate this, we emphasized reflexivity and member-checking throughout the sessions. This study allowed us to delve into the subjectivities and lived contexts of participants in rural areas of Ecuador. We followed the Consolidated Criteria for Reporting Qualitative Studies (COREQ) to develop to guide the development of this report [21].

### 2.2. Theoretical Perspective

This research draws on Bronfenbrenner’s Social Ecological Theory [22], which posits that the dynamic interaction between an individual and their environment shapes their behavior [23]. Specifically, we focus on the microsystem, in which the family serves as the immediate environment that influences adolescents. In rural communities, this framework highlights how parent-adolescent interactions underscore the critical role in shaping mental health outcomes.

Bronfenbrenner’s Social Ecological Theory was particularly appropriate for this study because it situates adolescent well-being within a nested system of environmental influences, with the family microsystem playing a foundational role. Recent evidence confirms the theory’s relevance in rural and low-resource settings. For instance, Tong and An [24] reviewed international applications of Bronfenbrenner’s model and emphasized its utility in intercultural education and development research; while Newland and Mourlam [25] applied the ecological model to examine rural children’s well-being during the COVID-19 pandemic. These recent studies support our theoretical framework and justify its selection for interpreting family functioning in adolescent mental health.

Group reflections with parents revealed that fostering healthy family interactions and providing emotional support were essential to promoting adolescents’ mental well-being. To enrich the analysis, we integrated the Calgary Family Assessment Model (CFAM) [26]. Its functional category enabled systematic assessment of expressive, relational, and supportive dimensions of family life. A recent systematic review by Mileski, McClay [27] confirms CFAM’s effectiveness in understanding family dynamics and supporting family-centered care in nursing contexts [28].

### 2.3. Study Participants

The study was performed in the Loja province, specifically in three rural communities: Guara, Chaquizhca, and Bellamaria. These communities are in the southern Andean region of Ecuador. A total of 29 parents of adolescents participated in the five FGDs. Participants were recruited through community leaders, who invited parents to attend an open meeting. Participation was voluntary, and no incentives were provided. While recruitment was non-probabilistic and based on convenience, efforts were made to ensure the inclusion of both mothers and fathers across the three communities in sparsely populated parishes with limited transport. We acknowledge that this approach may limit representativeness and introduce selection bias, which is a common limitation in qualitative PAR.

However, the sample size was adequate under the information-power principle, given the study’s narrow aim, the homogeneous participant role, dialogue-rich data augmented by Photovoice, and a focused analytic framework. Code saturation was reached by the fourth FGD, and meaning saturation was elaborated in the fifth.

### 2.4. Inclusion Criteria

Parents of adolescents residing in the participating rural communities who were willing to participate in the study.

### 2.5. Exclusion Criteria

Parents were excluded if they did not meet the inclusion criterion (being the parent of an adolescent in the participating communities) or were unable to participate in FGDs due to scheduling conflicts. Signed informed consent was required for participation.

### 2.6. Data Collection in FGDs and Photovoice

Data were collected in June 2022 as part of the Tropical Disease Research and Service-Learning Program (TDR-2022), a collaboration between Ohio University and the Pontifical Catholic University of Ecuador. This program is distinguished by its transdisciplinary approach. Initial contact was established through community leaders from the three participating rural communities, who coordinated the date and time for the first meeting with parents of adolescents. During this meeting, the research team presented the project’s objectives, participation guidelines, and ethical considerations, emphasizing the voluntary and non-remunerated nature of participation.

### 2.7. FGD Procedure

A moderator and three non-participant observers led the research team, allowing them to enrich the construction of the analyses with the information obtained. The activities were conducted in schools, with permission from community members who also hosted some meetings in their homes.

### 2.8. Photovoice Procedure

During the first meeting, parents of adolescents received instruction and training about Photovoice. Through pictures, they illustrated their personal strengths and the protective factors that families can implement to foster mental health in adolescents. The meaning of the photos taken was discussed and selected by participants during the group discussion.

### 2.9. Interview Guide

The research team designed a guide to implement the FGDs in the field, detailing the roles of the moderators and observers. The questions that guided the discussion were based on Bronfenbrenner’s ecological systems theory [16]. The initial question was as follows: What are your opinions regarding the family strengths that offer protection to adolescents? Additionally, participants took and analyzed photographs that represent the most relevant protective factors from their perspective. Discussions in each group lasted between 30 and 60 min, with an additional average of 15 min for the photo-taking process.

Regarding demographic characteristics, participants’ ages ranged from 26 to 47 years, with more than half being female. In terms of occupation, most men reported working as farmers, while most women were homemakers.

### 2.10. Data Analysis

Audio files were transcribed verbatim, anonymized, and managed/analyzed in ATLAS.ti. We combined inductive categorization with an integration approach informed by qualitative content analysis [29].

Coding process. Two researchers independently coded the first two transcripts line by line, developed a provisional codebook, and reconciled definitions in consensus meetings (with a senior qualitative lead available for arbitration). The refined codebook was then applied to the remaining transcripts and Photovoice narratives using constant comparison and reflexive thinking.

Intercoder agreement and credibility. We adopted a negotiated-agreement approach that emphasized analyst triangulation and structured consensus rather than relying on a single reliability coefficient. Double-coded segments were compared in ATLAS.ti (Code–Document and Co-occurrence tables), discrepancies were discussed, and decisions were documented in an audit trail. Credibility was further supported through member checking during FGDs and data triangulation (FGDs + Photovoice).

Category integration. Conceptually proximate codes were collapsed into subcategories and organized around a core category, or super-code (Table 1). A core category was retained if it (i) appeared across all data sources and (ii) was connected to every subcategory through at least one relation along the analytic chain (attributes → skills → family environment).

We then created a structural network in ATLAS.ti to make these relations explicit. In this network, solid black arrows labeled *is associated with* indicate robust, recurrent lateral links between subcategories, and solid black arrows labeled *is part of* denote hierarchical membership; both types were observed across multiple groups and supported by co-occurrence evidence. Red dashed arrows are a project-specific visual cue emphasizing the super-code’s integrative role by pointing from each subcategory to the core category; they are not a distinct relation type and do not replace membership.

Photovoice integration: Photographs and captions were coded using the same codebook, and their convergence with FGD categories was examined. The emergent subcategories identified through Photovoice (e.g., family cohesion, hope, and resilience) were mapped in the final network. Likewise, citations corresponding to photographs were imported and visualized as nodes connected to their related subcategories, making their contribution explicit within the structural model.

### 2.11. Rigor and Quality Guarantee

To ensure the trustworthiness of the study, several strategies were implemented during data collection and analysis. First, moderators engaged in member checking throughout the FGDs by summarizing and paraphrasing participants’ responses to confirm accuracy and understanding. This approach validated interpretations in real time. Second, data triangulation was employed during analysis by comparing information across multiple FGDs and integrating Photovoice images with the narrative data. This process enhanced both the depth and credibility of the findings. Finally, the interdisciplinary composition of the research team strengthened the interpretive process by incorporating diverse professional perspectives and minimizing individual biases. Together, these strategies addressed credibility and contributed to the overall rigor of the study.

### 2.12. Ethical and Legal Aspects of the Study

The study protocol followed the ethical principles of the Declaration of Helsinki. The research was approved by the Human Research Ethics Committee of the Pontifical Catholic University of Ecuador under Protocol No. EO-89-2022.

## 3. Results

**Figure 1:**
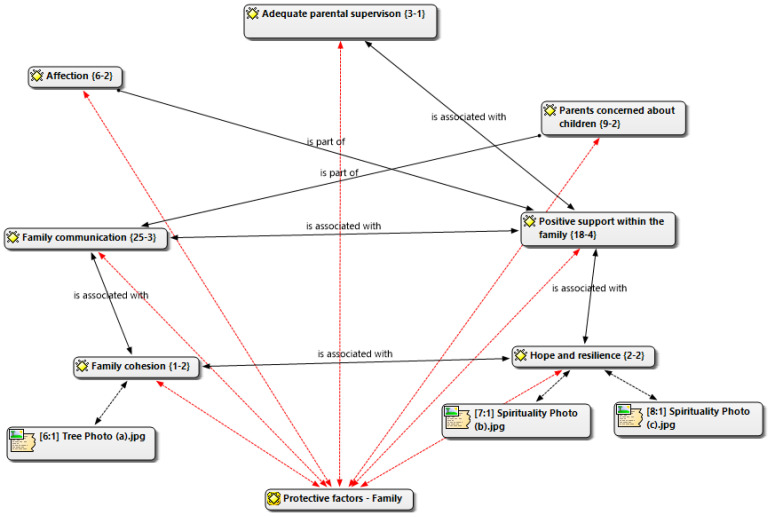
Structural network in ATLAS.ti. Solid black arrows labeled “is associated with” indicate robust, recurrent lateral links between subcategories, and solid black arrows labeled “is part of” denote hierarchical membership links; both types were observed across multiple groups and supported by co-occurrence evidence. Red dashed arrows are a project-specific visual cue underscoring the super-code’s integrative role by pointing from each subcategory to the core category. In addition, citations corresponding to Photovoice images were imported into the network and appear as nodes linked to the relevant subcategories, making their contribution explicit in the structural model.

Across five focus-group transcripts and the Photovoice artifacts, we identified one deductive anchor (Protective factors—Family) and seven inductive subcategories. Figure 1 displays the five most salient subcategories to illustrate the integration pattern around the super-code. We present results by (a) groundedness (how often participants referred to each subcategory) and (b) theoretical density (the number and strength of its links to other subcategories and to the super-code).

### 3.1. Family Strengths as a Protective Factor

Across five focus-group transcripts and the Photovoice artifacts, we identified one deductive anchor (Protective factors—Family) and seven inductively derived subcategories. Figure 1 presents the five most salient subcategories to illustrate the integration pattern around the super-code. Results are presented according to (a) groundedness—how often participants referred to each subcategory—and (b) theoretical density—the number and strength of its links to other subcategories and to the super-code (Figure 1).

### 3.2. Positive Support Within the Family

In this subcategory, parents expressed their views on positive support for the physical, emotional, and social development of their children during adolescence. Some of the expressions included in the subcategory ‘affection’ are associated with positive support, which forms part of the family’s emotional function. Love and acceptance dominate the discourse in this subcategory.

“I think it would be having a little patience and affection. We must be tolerant and teach them. As one is older it is like giving to the youngest, giving him more affection.” (SP6-FG4).

“To be loved, to be listened to, to be helped or not to get too angry.” (SP2-FG2).

The following excerpts indicate that the value of positive support emphasizes the protective function of the family, which involves meeting basic needs through economic support, alongside the socializing function, which is characterized by guidance and recommendations that foster healthy interpersonal relationships and encourage discipline.

“Adolescents will be happier if there is understanding at home and if they are helped with whatever they need.” (SP5-FG1)

“It is the part that we must give to our children: to talk, advise, and help.” (SP1-FG4)

“If we support them through thick and thin, with advice or money, they will move forward. That is why it is good to support them. And if they are not supported, then they will fall behind in their studies. And studying is the main thing, because now without studying they are nothing.” (SP4-FG4)

### 3.3. Adequate Parental Supervision

The subcategory ’adequate parental supervision’ reflects parents’ everyday experiences and their shared search for ways to maintain discipline and meet family expectations. This subcategory also identifies the family’s role in socialization, which explains its link in the structural network to the subcategory ‘positive support within the family’. The first testimony in the FGDs sparked a discussion about the crucial role of family–school collaboration in ensuring adequate supervision:

“They didn’t want anything (teenagers), they didn’t want to do their schoolwork, that’s why they lost the year [did not advance to the next school grade]. So, when I recovered, I went to talk to his teachers to find out what was happening. All my children attended the same school, so the teachers already knew me, but when these two missed the year [failed the grade], I explained my health situation, everything I went through, and they told me that we should try to have more communication. They did not know them.” (S6-FG4).

In another FGD, adequate parental supervision emphasized the importance of guidance focused on adolescent development. “Knowing what adolescents do” fosters interdependence in family functioning that ultimately supports adolescents’ independence in adulthood:

“Encourage them to study, know what they are doing, so they do not get married so quickly.” (SP3-FG5).

Both perspectives from the FGDs underscore the socialization function of the family as a protective factor, supporting adolescents’ adaptation to family life and the construction of their social identity.

### 3.4. Family Communication

The subcategory ‘Family communication’ indicates, from parents’ experiences, the importance of this practice for healthy coexistence and unity among family members. One of the stories illustrates the potential of family routines and daily activities to promote communication:

“Family dialogue is positive.” (SP1-FG1).

“Communication, talking to children.” (SP2-FG2).

“You always must talk with them. It is necessary communication between parents and adolescents”. (SP-FG3).

“I always have the idea in my home that at the table we eat, in the morning or at night, I talk to my children about their things.” (SP5-FG4).

“Now I think about the home, the family, it is always the dialogue for the children and the dialogue for the home, because if you go the hard way, you will not achieve anything like that in a short time.” (SP2-FG4).

The subcategory ‘Parents concerned about children’ is nested within the broader category ‘family communication’. According to participants’ experiences, these concerns referred to the difficulties or perceived needs that arise in interactions with adolescents, as well as the coping strategies parents use to address everyday challenges. This subcategory is linked to ’family communication’ because dialogue was the primary strategy parents reported using to strengthen their bond with their children:

“Sometimes families suffer from things that happen to teenagers.” (SP5-FG3).

“When there is trust, the child can tell his father or mother. I think that there one could realize what is happening with them to give them advice.” (SP1-FG5).

“When I see them, I always ask them what’s happening? In health problems more than anything or I insist that they tell us the other things that happen to them. Then they say yes, mom, I’m missing something. So, I say don’t worry, we’ll figure it out.” (SP6-FG4).

### 3.5. Photovoice Component

After the FGDs, participants took photographs and reflected on the meaning of the images. Together with the research team, they discussed the most significant family factors that protect adolescent mental health.

**Figure 2:**
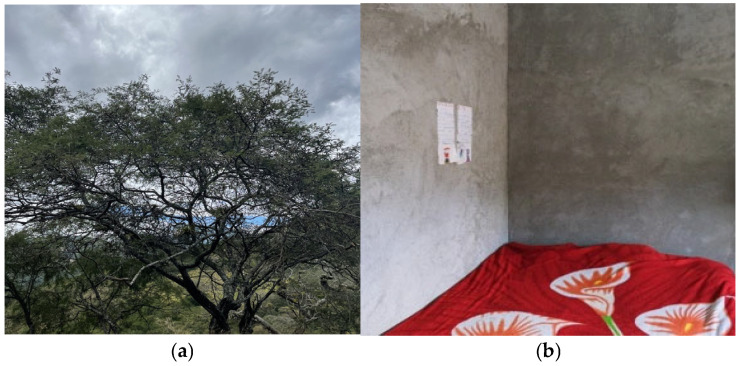

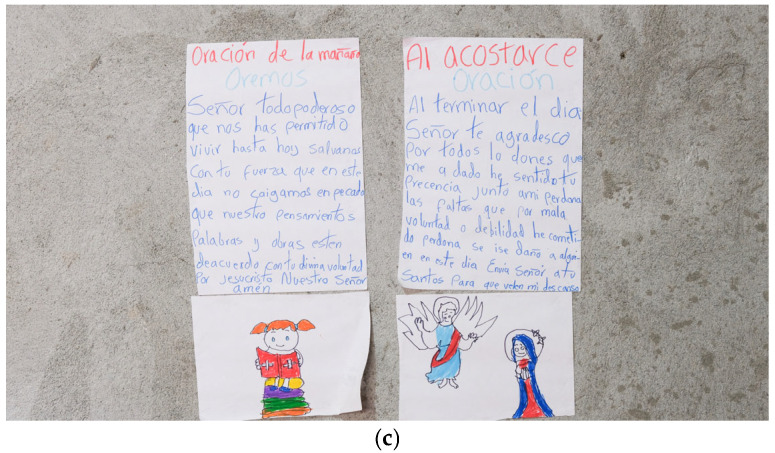
(**a**) A tree; (**b**,**c**) spirituality. Source: FGDs and pictures selected by participants.

From the parents’ perspective, picture (a), a tree, represented the adolescents of the community and symbolized the path of life (Figure 2). At different moments, decisions are made about what is right or wrong, but the most important elements are care, support, and family cohesion. This photograph was subcategorized under ’family cohesion’ and was also associated with the subcategory ’family communication’ since participants emphasized dialogue and interaction as essential for maintaining that cohesion.

Pictures (b) and (c), spirituality, gave rise to the emergent subcategory ’hope and resilience’, which was associated with the subcategory ’positive support within the family’ because prayer and faith practices were described as daily sources of encouragement and emotional support (Figure 2). These photographs depict a place at home used for prayer, where children each morning and evening ask for protection, express gratitude, and seek forgiveness. For parents, spirituality provided an additional source of motivation to persevere through each day.

Our interpretation of these subcategories is that a tree and spirituality represented key family strengths that promote adolescent mental health. Both serve as powerful elements of resilience for parents of adolescents in rural areas. This stage of the research highlighted that families’ primary goals in their interactions with adolescents were emotional support and socialization. For parents, a tree symbolized care, connection, and shared activities that keep the family united, while spirituality was rooted in religious practices and shaped their capacity to cope with the challenges of daily life.

## 4. Discussion

This study explored family functioning as a protective factor for adolescent mental health from the perspective of parents in rural communities of southern Ecuador, using Photovoice as a participatory research technique, and extends our 2023 risk-factor analysis by focusing on protective family dynamics, adding new knowledge about resilience pathways that can guide family-centered interventions. The methodology allowed parents to reflect on issues of family functioning and mental health that are rarely addressed in rural settings. Through photographs and FGDs, parents commented on aspects of family life that they perceived as necessary for supporting adolescents, such as effective communication, cohesion, supervision, and expressions of care. Our findings align with prior research showing that participatory methods such as Photovoice can foster collaboration, empowerment, and the inclusion of lived experience [30,31,32].

The images of the environment and symbolic objects captured in the photographs led to collaborative reflection between the research team and parents. Narratives generated in the FGDs motivated parents to consider ways of representing family functioning and how these practices might support their adolescents’ mental health. The main themes were related to caring. Previous studies highlight that Photovoice provides an innovative platform for collaboration and advocacy [33], promotes individual empowerment [34], and emphasizes inclusivity while valuing knowledge derived from lived experiences [35].

Most parents described behaviors they considered important for maintaining healthy family relationships, specifically protective, socializing, and nurturing functions. In our study, some parents reconstructed their family routines, offering insights into how families organize daily interactions and the care of adolescents. A quantitative study by Fosco and Lydon-Staley in 2020 [36] on family cohesion and conflict showed that everyday variations in these dynamics significantly impact adolescent mood and well-being. Other research has found higher mean scores in psychological well-being and emotional intelligence in families with high-functioning dynamics compared to those with moderate or severely dysfunctional functioning, underscoring the significance of the family environment in adolescent development [37].

Theoretically, within the family, expressiveness is linked to the affective function, encompassing both verbal and non-verbal emotional communication [27,38]. This study revealed that affection was predominantly manifested through expressions of care, concern, protection, or appropriate parental monitoring. Parents indicated, though to a lesser degree, the expression of affection through verbal affirmations or physical gestures such as embraces, implying that such practices are less common among rural households. Although such affective expressions were not common across all participants and FGDs, the discussions created opportunities to educate and expand awareness of the importance of this function through social interaction. According to the literature, parental warmth or affection represents a unique relational process that is essential for core developmental outcomes, including parent–child attachment, socialization, emotion recognition, responsivity, and empathy development [39]. Strengthening parental emotional warmth, perceived social support, and prosocial behavior among rural adolescents could effectively foster their sense of hope [40].

Spirituality also emerged as a common practice among families in rural communities. During the discussions, parents emphasized the importance of spiritual practices, representing them in the Photovoice activity with two pictures. Participants described spirituality as a protective factor that supports adolescents’ mental health. Prior studies have shown that positive parenting practices and effective parent–child communication are associated with higher spirituality [41]. Moreover, cultural values moderate the impact of parenting styles on child outcomes, supporting spiritual development. This interpretation is consistent with cross-cultural findings by Riany et al. [42], who demonstrated that although parental mood and parenting styles predicted child emotional and behavioral adjustment in both Australian and Indonesian samples, the expression and impact of these parenting practices differed across cultural contexts.

The spirituality and symbolic images in our data are consistent with the cultural specificity of rural southern Ecuador, where community life often integrates household, agricultural, and faith-based practices. Participants’ photographs functioned as symbolic anchors for family values and caregiving routines, helping parents articulate how daily practices of care, mutual support, and prayer are woven into family functioning. In this context, spirituality was described less as doctrine and more as a relational practice. The prominence of landscapes and objects in Photovoice further reflects how place-based meanings (home, farm, school, chapel, pathways) organize responsibilities and rhythms that parents perceive as protective for adolescents.

Promoting adolescent mental health remains a challenge for health systems worldwide due to the high burden of mental disorders. The inclusion of adolescent mental health as a component of the Sustainable Development Goals (SDGs), specifically SDG target 3.4, recognizes this field as a global priority [43,44]. Tamambang, Kusi-Mensah [45] further show that good mental health in children acts as a protective accelerator across multiple SDG targets, including reduced substance use and violence, and increased school performance. A systematic analysis of the global number of disability-adjusted life years (DALYs) revealed 125.3 million people affected [46]. LMICs are disproportionately affected due to shortages of mental health professionals and limited services in rural areas [47]. Moreover, parents in rural areas face significant challenges in mental health promotion, including the lack of structured educational programs that support families and prevent cognitive distortions—for example, perceptions of mental health topics as a weakness or of lesser importance [15].

Adolescence is a critical developmental period. Therefore, community and family nurses play an essential role in promoting healthy family relationships in rural settings. The specific gap addressed through the implementation of FGDs and Photovoice was the lack of health education on this topic. Using Photovoice, the research team built on the family experiences shared by participants through pictures, discussions, and reflections to elicit insights about protective factors for adolescent mental health and family functioning. This approach demonstrated cultural sensitivity and acceptability among participants. The central educational message was that good family functioning is closely related to good mental health. Our findings indicate that achieving positive relationships within families—particularly through cohesion and affection—contributes significantly to adolescents’ sense of control over their health and reduces the likelihood of mental health problems such as depression and anxiety [48]. Family cohesion, including daily interactions and family-centered activities, directly correlates with fewer depressive and anxiety symptoms [49].

This study presents several limitations. First, the small sample (*n* = 29 across five FGDs) and convenience recruitment via community leaders may have introduced selection bias toward parents who were more available or engaged; thus, the sample may not reflect the full range of parental perspectives in the area. Second, the findings are context-specific to rural southern Ecuador and are not statistically generalizable because our aim was analytic depth and transferability. Third, as with all group-based methods, social desirability and group dynamics may have influenced disclosures. Finally, the number of verbatim excerpts we could reproduce was constrained by confidentiality in small communities and variable audio quality. We partially mitigated these issues through member checking during FGDs, analyst triangulation with negotiated consensus, and data triangulation (FGDs + Photovoice).

## 5. Conclusions

This study demonstrated the important role of family strengths in protecting adolescent mental health in rural communities of southern Ecuador. Through participatory approaches such as Photovoice, families provided profound insights, emphasizing that effective communication, emotional support, parental supervision, and family cohesion serve as key pillars of resilience. Importantly, these qualities are not abstract concepts but dynamic, lived realities that help families navigate the complex challenges of economic hardship and geographic isolation. Nevertheless, these protective factors have their limitations, underscoring the vulnerability of support networks in resource-constrained settings.

The implementation of Photovoice converted participants from research subjects to collaborators, allowing them to critically reflect on their behaviors while aiding or reinforcing in the co-creation of strategies. This dual impact revealed protective factors and enabled families to regain control over their narratives and mental health outcomes. Moreover, the cultural and spiritual aspects highlighted in this study—especially the influence of faith and common values—demonstrated the significant interaction between belief systems and resilience. We frame our results as transferable to contexts where family life, faith practices, and place-based routines are intertwined, inviting readers to assess fit with local knowledge and service-delivery conditions. These insights may inform work in some rural LMIC settings with similar socio-cultural features, but require local adaptation and feasibility testing before broader application.

## Figures and Tables

**Table 1:** Description of family strengths category.

Deductive Category Assignments	Definition	Anchor Examples
Family strengths as a protective factor	Positive attitudes, values or beliefs. Conflict resolution skills. Good mental, physical, spiritual and emotional health.	‘Having effective communication with the children’; ‘being attentive to their needs’; ‘giving them love’.

Source: Analysis from the discussion of researchers.

## Data Availability

Data are not available [transcription] due to ethical restrictions.

## Abbreviations

The following abbreviations are used in this manuscript:

FGDs	Five Focus Group Discussions
LMIC	Low- and Middle-Income Countries
SDGs	Sustainable Development Goals
